# Interventions to Enhance Interoception and Symptom Attribution in Patients with Heart Failure: A Scoping Review

**DOI:** 10.1007/s11897-025-00733-w

**Published:** 2025-12-22

**Authors:** Michelle M. van Rijn, Tiny Jaarsma, Heleen Westland

**Affiliations:** 1 Julius Center for Health Sciences and Primary Care, Department of General Practice and Nursing Science, University Medical Centre Utrecht, Universiteitsweg 100, Utrecht, The Netherlands; 2 Department of Cardiology, University Medical Centre Utrecht, Utrecht, The Netherlands; 3 Department of Health, Medicine and Caring Sciences, Linköping University, Linköping, Sweden

**Keywords:** (4–6): Interoception, Symptom attribution, Heart failure, Interventions, Self-care, Symptom management

## Abstract

**Purpose of Review:**

Timely recognition and interpretation of symptoms are crucial for effective self-care in heart failure (HF). However, many patients struggle to detect bodily sensations and interpret their meaning, leading to poor outcomes. Interoception and symptom attribution are central to this process but are often impaired. This scoping review aims to describe interventions and their characteristics aiming to enhance interoception and symptom attribution in patients with HF.

**Recent Findings:**

Recent findings indicate that patients’ attributions of the causes of their symptoms strongly influence self-care, and growing attention to interoception suggests that increased awareness of bodily sensations can improve self-care symptom management.

**Summary:**

Interventions targeting interoception and symptom attribution may help improving HF patients’ symptom perception and self-care symptom management. Both constructs are closely connected and contribute to timely symptom management. The heterogeneity of interventions reflects the complexity of symptom perception in HF and highlights the need for personalized, multicomponent approaches to strengthen self-care and improve outcomes.

## Introduction

Heart failure (HF) is a chronic progressive condition, and most patients experience symptoms that affect their daily lives and quality of life [1, 2]. The impact of HF on patients’ life is substantial, as they are expected to adhere to medication, lifestyle changes, and self-monitor and address symptoms to prevent worsening of HF and hospitalization [3]. Therefore, self-care is one of the cornerstones of HF management and is associated with improved HF outcomes such as reducing hospitalizations, decreasing morbidity and mortality, and better well-being [4, 5]. According to the middle-range theory of self-care of chronic illness, self-care is a decision-making process that involves strategies for maintaining health through health-promoting practices and managing illness [6, 7]. Self-care encompasses three interrelated key components: self-care maintenance (behaviors to maintain stability), self-care monitoring (observing changes in signs and symptoms), and self-care management (responding to symptoms when they occur) [6]. To prevent symptom worsening, patients need to notice and interpret changes in their HF condition. Symptom perception is therefore an important process in HF self-care monitoring and self-care management and refers to both the detection of bodily sensations of symptoms and the interpretation of their meaning [8]. Symptom perception includes body listening, monitoring signs, recognizing, interpreting, and labeling (assigning or determining meaning to sensations) of symptoms [8, 9]. However, many patients with HF struggle with symptom perception, making it difficult to detect symptoms in a timely manner and respond adequately [10]. 

HF symptoms, such as fatigue, shortness of breath, edema, and dyspnea, often vary in intensity and may initially present subtle, making them difficult to recognize and assess its severity [1, 11]. Patients may normalize symptoms by adapting daily routines or reducing responsibilities to minimize their occurrence, have symptoms that overlap with those of other chronic diseases, have limited health literacy, or experience psychological barriers such as anxiety or depression [12–14]. These factors can hinder their accurate symptom perception. Even when HF symptoms are perceived, patients often incorrectly assign or attribute them to other causes, such as psychological distress or other comorbid illnesses instead, rather than to HF [15–17]. Correct symptom attribution is important for timely care-seeking and taking appropriate self-care actions, especially during periods of worsening of HF [18, 19]. Adequate symptom perception and attribution requires from patients to sense and make sense of bodily sensations. The ability to sense bodily sensations relates to the concept of interoception, which refers to sensing, interpreting, and bringing together signals of internal bodily changes, both consciously and unconsciously [20–22]. Interoception includes three facets: interoceptive sensibility (natural tendency to focus on internal bodily sensations), interoceptive accuracy (ability to detect internal bodily sensations), and interoceptive awareness (how well a person’s confidence in their interoceptive abilities matches their actual accuracy, i.e. metacognitive awareness) [22]. Enhancing interoceptive abilities may improve patients’ capacity to detect early bodily changes, interpret their meaning as being symptoms, and attribute them correctly to HF [23]. 

Health care professionals play a key role in supporting patients in enhancing interoceptive skills and symptom knowledge. Interventions to enhance interoceptive skills have received growing attention, particularly in mental health and to some extent in chronic physical conditions [22, 24]. For example, mindfulness-based interventions in patients with cancer have shown to enhance awareness of bodily sensations, which in turn can help reduce psychological distress or symptoms such as fatigue [25, 26]. Building on this, interventions that support symptom perception and accurate symptom attribution may also strengthen timely self-care. Structured symptom monitoring, decision aids, and tailored education are examples that have shown promise in improving chronic disease management [27]. 

Since interoception and symptom attribution are closely linked to the process of symptom perception, interventions that focus on these concepts may improve self-care symptom management among patients with HF. However, little is known about interventions in HF populations including interoception and symptom attribution to support symptom perception. This scoping review aims to comprehensively map and explore such interventions and their characteristics intended to enhance interoception and symptom attribution in patients with HF to summarize current evidence, clarify approaches and identify research gaps.

## Methods

### Design

This scoping review was conducted following the methodological framework of Arksey and O’Malley with the following steps: identify relevant studies; select studies based on pre-defined inclusion- and exclusion criteria; chart the data; analyze, summarize and report results [28]. The Preferred Reporting Items for Systematic Reviews and Meta-analyses for Scoping Reviews (PRISMA-ScR) was used to guide the reporting [29]. The protocol of this scoping review is registered at the Open Science Framework (OSF) (10.17605/OSF.IO/N74TX). Ethical approval was not needed for this review of existing literature.

### Identify Relevant Studies

Studies were included if they reported on interventions related to interoception or symptom attribution in adult patients (≥ 18 years) with HF or other cardiovascular diseases. Other cardiovascular diseases were included because few studies were expected to focus solely on HF, while cardiovascular conditions (e.g., coronary artery disease, myocardial infarction or general cardiac populations) may still provide insights relevant to the HF population. In this scoping review, interventions are defined as structured activities, a program or strategy, delivered by a healthcare professional, system or tool that is intentionally designed to bring about change in knowledge, skills, perception or behavior [30]. To be included, an intervention had to target interoception, and/or symptom attribution, and provide a description of the intervention’s characteristics. Intervention studies or intervention study protocols published in peer reviewed international journals were considered for inclusion. We excluded studies that were targeted at informal caregivers, health care professionals, healthy participants, children, or participants with a primary focus on psychiatric disorders (e.g. schizophrenia or other severe mental illnesses). Furthermore, book chapters, letters to the editor, guidelines, websites, and studies without full-text availability were excluded. No publication date restrictions were applied, given the anticipated limited body of research on these specific topics.

Two search strategies were developed: one focusing on interoception, and one on symptom attribution. The literature search was conducted in the databases of PubMed, EMBASE, CINAHL, and PsycINFO in April 2025. Search strategies were developed and validated in collaboration with a medical librarian. The strategies included combined keywords and Medical Subject Headings (MeSH). Reference lists of included studies were hand searched to ensure complete inclusion of relevant studies (Table 1.) 

**Table 1:** Search string PubMed

Interoception
((((((interocept*[Title/Abstract]) OR (interoception[MeSH Terms])) OR (body-awareness[Title/Abstract])) OR (body-listening[Title/Abstract])) OR (body-conscious[Title/Abstract])) OR (self-perception[Title/Abstract])) AND ((((((((((((((((((((((heart failure[Title/Abstract]) OR (hf[Title/Abstract])) OR (congestive heart failure[Title/Abstract])) OR (CHF[Title/Abstract])) OR (cardiac failure[Title/Abstract])) OR (heart decompensation[Title/Abstract])) OR (myocardial failure[Title/Abstract])) OR (heart failure[MeSH Terms])) OR (“coronary artery disease“[Title/Abstract])) OR (“coronary arteriosclerosis“[Title/Abstract])) OR (“coronary arterioscleroses“[Title/Abstract])) OR (“angina pectoris“[Title/Abstract])) OR (“CAD“[Title/Abstract])) OR (“heart disease“[Title/Abstract])) OR (“myocardial infarction“[Title/Abstract])) OR (“unstable angina“[Title/Abstract])) OR (“angor pectoris“[Title/Abstract])) OR (“coronary thrombosis“[Title/Abstract])) OR (“acute coronary syndrome“[Title/Abstract])) OR (“myocardial ischemia“[Title/Abstract])) OR (“cardiovascular disease“[Title/Abstract])) OR (“myocardial ischaemia“[Title/Abstract]))
Attribution
((((attribution*) OR (reattribution*)) OR (AR)) AND ((((((((((((((“symptom management“[Title/Abstract]) OR (“symptom perception“[Title/Abstract])) OR (“’perceived symptom*“[Title/Abstract])) OR (“symptom recogni*“[Title/Abstract])) OR (“symptom aware*“[Title/Abstract])) OR (“symptom experience*“[Title/Abstract])) OR (“symptom assess*“[Title/Abstract])) OR (“symptom monitor*“[Title/Abstract])) OR (“symptom manag*“[Title/Abstract])) OR (“signs and symptoms“[Title/Abstract])) OR (self care[MeSH Terms])) OR (“self care“[Title/Abstract])) OR (“self-care“[Title/Abstract])) OR (“self-management“[Title/Abstract]) OR (self management[MeSH Terms]))) AND ((((((((((((((((((((((heart failure[Title/Abstract]) OR (hf[Title/Abstract])) OR (congestive heart failure[Title/Abstract])) OR (CHF[Title/Abstract])) OR (cardiac failure[Title/Abstract])) OR (heart decompensation[Title/Abstract])) OR (myocardial failure[Title/Abstract])) OR (heart failure[MeSH Terms])) OR (“coronary artery disease“[Title/Abstract])) OR (“coronary arteriosclerosis“[Title/Abstract])) OR (“coronary arterioscleroses“[Title/Abstract])) OR (“angina pectoris“[Title/Abstract])) OR (“CAD“[Title/Abstract])) OR (“heart disease“[Title/Abstract])) OR (“myocardial infarction“[Title/Abstract])) OR (“unstable angina“[Title/Abstract])) OR (“angor pectoris“[Title/Abstract])) OR (“coronary thrombosis“[Title/Abstract])) OR (“acute coronary syndrome“[Title/Abstract])) OR (“myocardial ischemia“[Title/Abstract])) OR (“cardiovascular disease“[Title/Abstract])) OR (“myocardial ischaemia“[Title/Abstract]))

### Study Selection

Study selection was performed using the web-based platform Rayyan [31]. First, duplicates were identified automatically in Rayyan and then manually verified and removed. Second, titles and abstracts of the remaining studies were independently screened by two researchers (MR and HW). Each study was categorized as ‘include’, ‘exclude’, or ‘maybe’. Discrepancies and studies labeled as ‘maybe’ were resolved through discussion.

Full-text screening of eligible studies was performed by MR and HW. Each full-text study was categorized as ‘include’, ‘exclude’, or ‘maybe’. Discrepancies and studies labeled as ‘maybe’ were resolved through discussion. If a study referred to a study protocol for the complete intervention description, then the study protocol was included in this scoping review.

### Charting Data

Data were extracted using a structured format in Microsoft Excel. We extracted the data on study characteristics (author, year, country, study design, population), and on intervention characteristics (author, year, approach, intervention, intervention content, setting, interventionist, mode of delivery, and intensity and dose of the intervention). Furthermore, studies targeting interoception were examined to determine which facet of interoception the intervention primarily addressed, i.e. interoceptive sensibility, interoceptive accuracy, or interoceptive awareness. A second researcher (HW) checked the extraction file and randomly checked 25% of the data extraction to ascertain accuracy and completeness of the extraction.

## Results

**Fig. 1:**
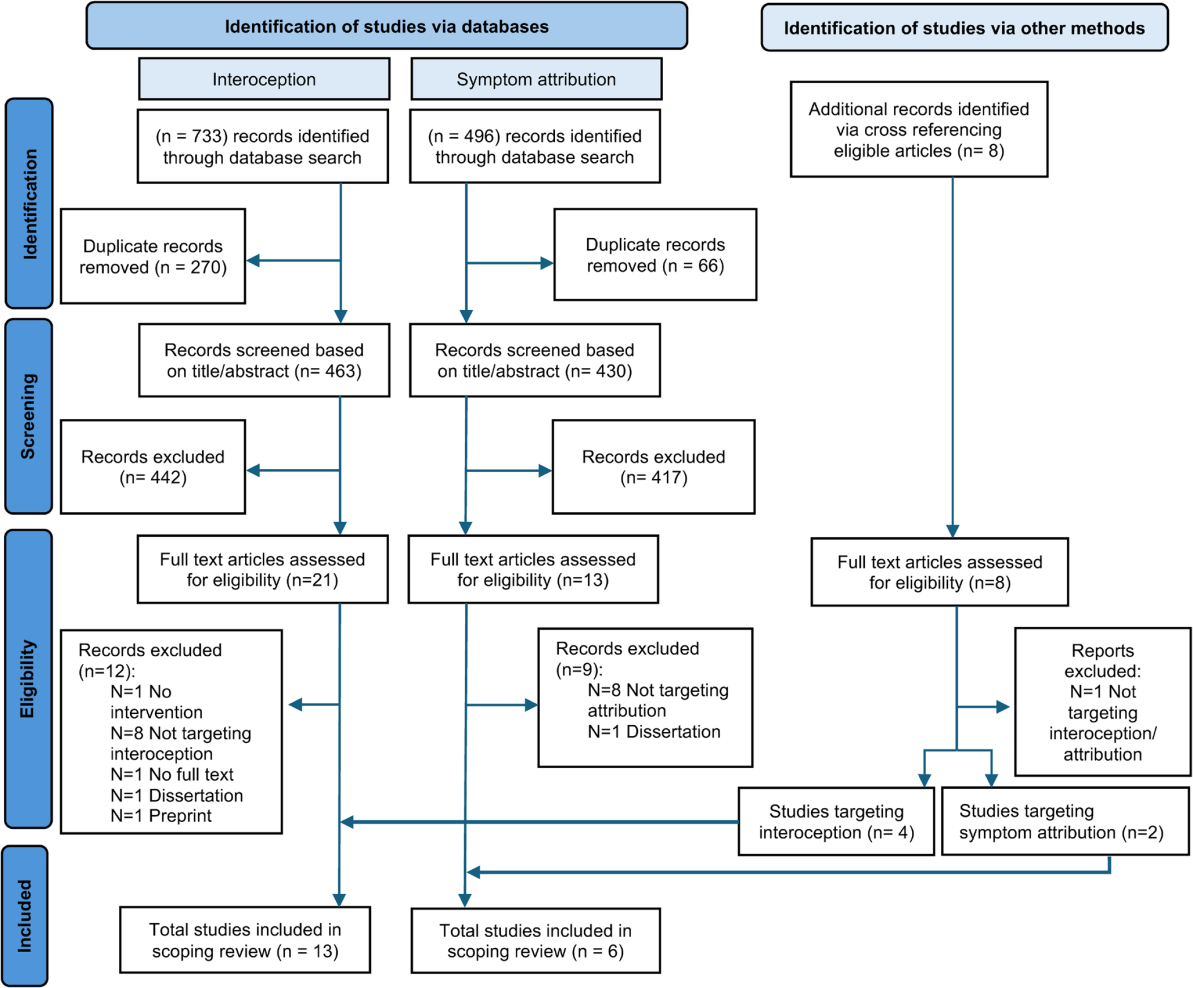
PRISMA Flowchart of the selection of studies of interventions enhancing interoception or symptom attribution

The search identified a total of 1229 records, of which 336 duplicates were removed. We identified and screened a total of 893 potential studies on title and abstract, of which 859 were excluded since they did not meet the inclusion criteria. The remaining 34 studies and a total of 8 hand searched studies were screened for full text, which resulted in inclusion of a total 19 studies that met the inclusion criteria, see Fig. 1.

### Study Characteristics

Studies addressing interoception (*n* = 13), were published between 2004 and 2024, with a substantial increase of published studies since 2019. Most studies were conducted in the United States (*n* = 8) [32–39] and had a randomized controlled trial (RCT) design (*n* = 6), followed by study protocols (*n* = 3). Ten studies specifically involved patients with HF. Intervention studies included both men and women.

Studies addressing symptom attribution (*n* = 6) were published between 1999 and 2020. Most studies were conducted in the USA (*n* = 3) [40–42] followed by the Netherlands (*n* = 2) [43, 44], with RCTs being the most used design (*n* = 5). Three studies focused on patients with HF, and three studies on patients with CVD more broadly such as patients with coronary heart disease or myocardial infarction [40]. (Table 2.)

**Table 2:** Characteristics of included studies

Author (year of publication)	Country	Study design	Study population condition^b^
Studies on interoception			
DeWalt et al. (2004) [37]	USA	Pretest/Posttest	HF
Farris et al. (2024) [32]	USA	Study protocol for RCT	CVD
Gentile et al. (2022) [45]	Canada	Pilot RCT	CVD (e.g. at risk for CAD)
Hoffmann et al. (2023) [46]	Germany	Cross-sectional observational comparison^a^	HF
Jurgens et al. (2013) [36]	USA	RCT	HF
Keirns et al. (2023) [34]	USA	Study protocol for RCT	HF
Loucks et al. (2023) [33]	USA	RCT	CVD (hypertension)
Matsuda et al. (2022) [47]	Japan	RCT	HF
Salmoirago-Blotcher et al. (2012) [38]	USA	Study protocol for pilot RCT	HF
Salmoirago-Blotcher et al. (2022) [35]	USA	Pretest/Posttest	HF
Pereira Sousa et al. (2021) [48]	Portugal	Pilot RCT	HF
Sullivan et al. (2009) [39]	USA	Prospective experimental cohort	HF
Teng et al. (2018) [45]	Taiwan	RCT	HF
Studies on symptom attribution			
Engelen et al. (2020) [44]	Netherlands	Pilot RCT	CVD (e.g. MI)
Lorig et al. (1999) [41]	USA	RCT	CVD (e.g. heart disease)
McKinley et al. (2009) [40]	USA	RCT	CVD (e.g. coronary heart disease)
Meng et al. (2013) [49]	Germany	Study protocol for cluster RCT	HF
Nundy et al. (2013) [42]	USA	Pretest/Posttest	HF
Smeulders et al. (2010) [43]	Netherlands	RCT	HF

*RCT* randomized controlled trial, *HF* heart failure, *CVD* cardiovascular disease, *MI* Myocardial infarction, *CAD* coronary artery disease

^a^ Study design assigned by the authors of the scoping review based on information found in the study.

^b^ Condition categorizations of study population assigned by the authors of the scoping review based on information found in the study.

### Characteristics of Interventions on Interoception

The interventions on interoception varied in intervention approach, including mindfulness-based (*n* = 7), educational (*n* = 3) and behavioral (*n* = 3) interventions. Mindfulness-based interventions aimed to enhance interoception through practices such as breathing techniques, body scan, mindful movement, open-awareness meditation [34, 35, 38, 45], and full mindfulness-based stress reduction (MBSR) programs [33, 39, 50]. Most of these interventions were delivered face-to-face either individually or in groups, although three studies were delivered by telephone. Interventions were led by psychologists or instructors specialized in mindfulness or MBSR. Duration ranged from structured 8-week programs to shorter sessions supplemented with daily home practices. Intervention intensity per session varied from 30 min to 2.5 hours.

Educational interventions focused on improving symptom recognition and response behaviors, often as part of broader programs [36, 37, 48]. These interventions were typically delivered in individual, face-to-face sessions by principal investigators, health educators, or nurses, and were supported by printed materials such as booklets. Intervention intensity ranged from a single 20–30 min session [36] to a one-hour session followed by 7 follow-up calls [37]. 

Behavioral interventions employed techniques to enhance bodily sensations and symptom interpretation, including heartbeat tracking [46], use of wearable technology [47], and exposure-based approaches [32]. All interventions were delivered individually and face-to-face, by clinical psychology students or a research nurse. One study did not report who delivered the intervention [46]. Intervention intensity varied from a single session to a month-long intervention, with individual sessions lasting 30–60 min (see Table 3). The studies also varied in how they addressed interoception. Interoception was included as a primary outcome or as a process targeted by the intervention to enhance patient’s ability to detect and interpret internal bodily sensations. Five studies explicitly specified the interoceptive facet. One study addressed interoceptive accuracy solely using the heartbeat tracking task [46], while another study targeted both interoceptive awareness and accuracy, combining the heartbeat tracking task with an interoceptive awareness questionnaire [34]. Three studies focused solely on interoceptive awareness, using an interoceptive awareness questionnaire to measure interoception [33, 35, 45]. For the remaining eight studies, the interoceptive facet was not clearly specified by the authors. In these studies, the intervention was therefore categorized to the most appropriate facet(s) based on its characteristics and objectives. Four of these studies primarily focused on interoceptive sensibility, aiming to enhance patients’ subjective perception and attention to bodily sensations [36, 37, 45, 47]. Four studies mainly addressed interoceptive awareness by using mindfulness practices [32, 38, 39, 50]. None of these studies combined the interoceptive facets (Table 4).

**Table 3:** Study intervention and characteristics of included studies

Author (year)	Intervention approach	Intervention	Intervention content	Setting	Interventionist	Mode of delivery	Intensity & dose
Studies on interoception							
DeWalt et al. (2004) [37]	Educational	Disease management program	• Education session included: review booklet, went over management scenarios • Booklet included: explanation HF, symptoms, signs of worsening, salt avoidance, medication compliance, weight change, daily assessment of weight and edema, titrating diuretics instructions, instructions on contact disease management team, plans to implement self-care strategy • Follow-up calls to reinforce educational session and to troubleshoot diuretic dose adjustment.	Outpatient (university internal medicine clinic)	Pharmacist/health educator	Individual, face to face	• Intensity: 1 h educational session. Follow-up calls: 5–15 min • Dose: educational session once, follow-up calls spread over 12 weeks
Farris et al. (2024) [32]	Behavioral	Theoretically informed cognitive-behavioral intervention	• Sessions included: psychoeducation, setting PA goals, rational for exposure/fading safety behaviors, develop exposure hierarchy, mindfulness, exposure practice, fading safety behaviors, goal setting, planning for maintenance of PA • Patients monitor their PA and receive feedback during each session	Center-based (CR location)	Clinical psychology doctoral students	Individual, face to face	• Intensity; 6 sessions of 45–60 min • Dose: sessions 2x/week for 3 weeks
Gentile et al. (2022) [50]	Mindfulness based	MBSR program	• Sessions included: meditation teacher instructions, and group discussions. • Retreat session: further integrate acquired skills • Homework: practice formal mindfulness skills and record minutes of daily practice	Center-based (community-center)	Psychologist/MBSR teacher	Group, face to face	• Intensity: 2.5 h-sessions, 5 h retreat, 25–45 min homework • Dose: 8 weekly sessions, one-time retreat, daily homework
Hoffmann et al. (2023) [46]	Behavioral	Heartbeat tracking	• Heartbeat tracking task: count heartbeats without manually checking pulse while visual signal indicates start and end of counting period.	Center-based (university)	Not specified	Individual, face to face	• Intensity: 4 heartbeat tracking trials using time intervals of 25 s, 35 s, 45 s, and 55s. • Dose: on one day
Jurgens et al. (2013) [36]	Educational	HF symptom monitoring awareness & response training	• HF self-care booklet • Intervention components: somatic awareness, daily symptom graph, track symptoms and responses, symptom profile, symptoms at rest, respiratory effort and rate symptoms, meaning of symptoms and response, • Home visit: to review training	Home-based	Principal Investigator	Individual, face to face	• Intensity 20–30 min • Dose: not specified
Keirns et al. (2023) [34]	Mindfulness based	Mindfulness training	• Sessions included: Awareness of breath, body scan, training to direct attention to activities of daily life, awareness of thoughts and emotions, practice open awareness • Home practice: guidance of digital mindfulness recording	Home-based	Mindfulness training instructor	Individual, via telephone	• Intensity: 30-minute sessions, 20 min homework • Dose: 8 weekly sessions, daily home practice
Loucks et al. (2023) [33]	Mindfulness based	Mindfulness-based Blood Pressure Reduction program	• Orientation session included: personalized health feedback on HT risk factors, education on impact of HT • Group sessions included: introduction BCT & HT, mindful eating, pleasant events calendar, information & review of basic forms of PA, unpleasant events calendar, PA, Aerobic PA, motivational interviewing, BP goal setting, medication adherence, social support, theoretical mechanism for mindfulness on CVD • Retreat session: Aerobic PA, DASH lunch, self-care reflection • Recommended home mindfulness practices	Outpatient (university health center)	MBSR instructor	Group, face to face (partly digital due to Covid)	• Intensity: 2.5 h-group sessions, 7.5 h-retreat session, 45 min home practice • Dose: 8 weekly group sessions, 1-day retreat session, home practice 6 days per week
Matsuda et al. (2022) [47]	Behavioral	Self-monitoring support session	• Wristwatch • Briefing on: measured activity counts, sleep and wake time, activity and sedentary time, activity intensity. • Support session included: using personalized wristwatch data to reflect on and describe physical sensations to enhance symptom awareness	Outpatient (outpatient clinic)	Trained research nurse	Individual, face to face	• Intensity: wristwatch 24 h, 30-minute support session. • Dose: wristwatch 3–7 days post discharge and at 1 month follow-up, one-time support session
Salmoirago-Blotcher et al. (2012) [38]	Mindfulness based	Mindfulness training	• Sessions included: awareness of breath, sounds, emotions, and thoughts, open awareness, mindful eating and drinking, body scan • Home practice: audio CD with sitting- and body scan practice	Home-based	HCP/mindfulness professional training program graduates	Individual, via telephone	• Intensity: 30-minute telephone session, 2 × 20 min home practice • Dose: 8 telephone sessions, home practice daily at least once a day.
Salmoirago-Blotcher et al. (2022) [35]	Mindfulness based	Mindfulness training	• Sessions included: Attention-focusing and open-awareness practices • Home practice: digitally recorded guided mindfulness practice	Home-based	Certified instructors	Individual, via telephone	• Intensity: 30-minute sessions, 20-minute home practice. • Dose: 8 weekly sessions, daily home practice
Pereira Sousa et al. (2021) [48]	Educational	Fluid and self-care management program	• Leaflet: HF information, symptoms, awareness of detection, fluid management plan. Patients explain understanding of leaflet information. • Weight diary • Follow-up contact: Nurse address reinforcements on explanation HF signs and symptoms and recognition, importance of fluid management, instructions on contacting HCP	Home-based	HF nurse	Individual, face to face	• Intensity: not specified • Dose: follow-up 3 times
Sullivan et al. (2009) [39]	Mindfulness based	Mindfulness-based psychoeducational support	Group sessions included: • MBSR; body scan, awareness of breathing, loving kindness • Coping skills training; overview HF, HF treatments, diet and exercise, stress management, assertive communication, social support, learned optimism, healthy grieving, nondenominational spirituality and health • Expressive support group discussion; sharing emotional content and group affiliation • Workbook: to supplement learning and encourage self-reflection • Home practice: audio guided meditations	External and home-based	Mindfulness meditation instructors	Group, face to face	• Intensity: 2.25 h group sessions, 30-minute home practice • Dose: 8 weekly group sessions, daily home practice
Teng et al. (2018) [45]	Mindfulness based	Walking with breathing program	Program included: • Warming up: stretching exercises, teach abdominal deep breathing patterns. • Main course: walking with abdominal breathing patterns. • Cool-down: slowing walking speed, top walking, return to normal breathing rate. • Home practice: continue with WwB, exercise diary	Outpatient (hospital) and home-based	Researcher	Individual, face to face	• Intensity: 15 min home practice • Dose: 2x/day for 12 weeks
Studies on symptom attribution							
Engelen et al. (2020) [44]	Behavioral	Self-management program	• Modules included: coping with CVD, setting boundaries in life, lifestyle, healthy nutrition, physical activity in a healthy way, interaction with HCPs • Exercise and nutrition diary to register behavior in exercise and nutrition routines • Personalized and supported by written information, tailored feedback, quotes and video from other patients with CVD, pictures, and exercises.	Home-based	No interventionist (unguided)	Individual, digital	• Intensity: 3–4 sessions per module, no time specified • Dose: 12 months access to program with unlimited access
Lorig et al. (1999) [41]	Educational	Chronic disease self-management program	• Sessions included: exercise, use of cognitive symptom management techniques, nutrition, fatigue and sleep management, use of community resources, use of medications, dealing with emotions, communication with others, problem-solving, and decision-making. Content was taught using weekly action planning, feedback, modeling of behaviors, problem-solving by participants for one another, reinterpretation of symptoms by giving many possible causes for each symptom, management techniques, group problem-solving, decision-making.	Center-based (community-based sites)	Pair of trained volunteer lay leaders	Group, face to face	• Intensity: 2.5 h- sessions. • Dose: 7 weekly sessions
McKinley et al. (2009) [40]	Educational	Education and counseling	• Session included: information on symptoms, symptom presentation, symptom onset, appropriate actions, National Heart Attack Alert Program form, anticipating emotional responses to symptoms, rewards of seeking treatment quickly, addressing emotional issues through scenarios and role playing, consulting spouse or significant others in case of symptoms • Follow up call: to review main points from session.	Outpatient (clinic or research office) or home-based	Cardiovascular nurse	Individual, face to face	• Intensity: 40-minute session, 10–15 min follow-up call • Dose: one session, one follow-up call
Meng et al. (2013) [49]	Educational	Self-management educational program	• Booklets: HF information and symptom-monitoring diary for 12 months • Sessions included: HF illness, treatment knowledge (etiology, symptoms and signs, diagnostics, treatment options), self-management behaviors (diet, attention to signs and symptoms, daily weight, BP monitoring), medication adherence, promoting physical activity (using action and coping planning, self-monitoring), illness related problems, signs of emotional distress, coping strategies	Center-based (CR location)	Physician/nurse/psychologist/physiotherapist	Group, face to face	• Intensity: 60–75 min sessions • Dose: 5 sessions
Nundy et al. (2013) [42]	Educational	Message-based self-management intervention	• Text messages included: medication adherence, dietary compliance, appointment adherence, HF signs and symptom recognition, management if experiencing symptoms, health care navigation	Home-based	Text message communication platform for health researchers	Individual via telephone	• Intensity: text messages were personalized to patients’ medication and appointment regimen • Dose: 30 days
Smeulders et al. (2010) [43]	Educational	Chronic disease self-management program	• Sessions include: skills mastery with goal setting and action planning; reinterpreting symptoms with cognitive symptom management techniques, deal with relieving symptom problems; modelling and social persuasion through group participants/leaders, participants are expected to become motivated to change behaviors and beliefs	Outpatient (hospital)	Cardiac nurse specialist/patient with CHF	Group face to face	• Intensity: 2.5 h group session • Dose: 6 weekly group sessions

*PA* physical activity, *HT* hypertension, *BCT* behavior change theory, *BP* blood pressure, *CVD* cardiovascular disease, *DASH* dietary approaches to stop hypertension, *WwB* walking with controlled breathing, *ECG* electrocardiogram, *HF* heart failure, *HCP* health care professional, *MBSR* mindfulness based stress reduction, *ACS* acute coronary syndrome, *CR* cardiac rehabilitation, *CHF* chronic heart failure

**Table 4:** Studies on interoception and their focus on the interoceptive facets

Author (year)	Interoceptive sensibility	Interoceptive accuracy	Interoceptive awareness
DeWalt et al. (2004) [37]	X^a^		
Farris et al. (2024) [32]			X^a^
Gentile et al. (2022) [50]			X^a^
Hoffmann et al. (2023) [46]		X	
Jurgens et al. (2013) [36]	X^a^		
Keirns et al. (2023) [34]		X	X
Loucks et al. (2023) [33]			X
Matsuda et al. (2022) [47]	X^a^		
Salmoirago-Blotcher et al. (2012) [38]			X^a^
Salmoirago-Blotcher et al. (2022) [35]			X
Pereira Sousa et al. (2021) [48]	X^a^		
Sullivan et al. (2009) [39]			X^a^
Teng et al. (2018) [45]			X

^a^ Interoceptive facet assessed by the authors of the scoping review based on information found in the study

### Characteristics of Interventions on Symptom Attribution

The interventions on symptom attribution had either an educational (*n* = 5) or behavioral (*n* = 1) approach. Educational approaches aimed to enhance patients’ understanding and interpretation of symptoms. Techniques included discussing different meaning of symptoms, practicing decision-making, and working with example scenarios. The interventions were delivered face-to-face, with three studies conducted in group settings, and were supported by printed materials [41, 43, 49]. Interventionists were volunteer leaders, nurses, peer-patients with HF or a combination of facilitators. Intervention intensity ranged from a single 40-minute session to seven weekly sessions of 2.5 h. One intervention used an automated text messaging system (SMS) using the telephone, to support education. The intervention was delivered through a text message communication platform for health researchers, and the intensity of the SMS delivery was personalized to patients’ medication and appointment regimen [42]. 

One intervention used a behavioral approach through a web-based platform to support symptom monitoring, and treatment adherence remotely (Table 3) [44]. The platform consisted of modules with 3–4 sessions per module. The interventionist and intensity in time of this platform was not reported. However, participants had access to the digital program for 12 months and could use it as often as they wished [44]. 

Interventions focusing on symptom attribution (*n* = 6) aimed to improve patients’ ability to correctly interpret bodily symptoms in the context of HF or CVD. Educational components covered symptom recognition, variability in symptom presentation, and response behaviors [40, 49]. Other studies supported patients in rethinking or reinterpreting their symptoms to manage symptoms [41, 43]. Three studies also included elements of action planning and problem-solving strategies to help patients understand and respond to early warning signs of worsening HF [41, 43, 49]. 

## Discussion

This scoping review provides a comprehensive overview of interventions aimed at enhancing interoception and symptom attribution in patients with HF. Our findings indicate that, although this field is still emerging, a range of approaches and interventions was identified. Interoception was most often targeted through mindfulness interventions, whereas symptom attribution was primarily addressed with educational approaches.

Among the 13 studies focusing on interoception, we identified a considerable number of mindfulness-based approaches (*n* = 7) in their intervention. These interventions specifically aimed to enhance interoceptive awareness by training patients to attend to bodily sensations through practices such as meditation, body scans, and breathing techniques. This emphasis on interoceptive awareness reflects the way mindfulness is conceptualized in the literature. Mindfulness mainly helps patients to notice their bodily sensations and reflect on their meaning in a conscious and non-judgmental way, rather than improving the objective detection of internal sensations (interoceptive accuracy) [51–53]. Our findings show that mindfulness-based interventions largely contribute to interoceptive awareness but rarely address interoceptive sensibility or accuracy.

Educational approaches (*n* = 3) primarily focused on interoceptive sensibility, by encouraging patients to pay attention to and reflect on bodily cues. This suggests that educational formats may be suitable to strengthen patients’ subjective perception in focusing on interpreting bodily sensations, although this facet was not explicitly described by the authors and was therefore categorized as such in our review.

More variation in the interoceptive facet with behavioral approaches (*n* = 3) was observed. While one intervention explicitly targeted interoceptive accuracy, the others primarily addressed interoceptive awareness or interoceptive sensibility. This heterogeneity suggests that behavioral approaches vary in which interoceptive facets they target, more research can determine which facets are most effectively addressed with a behavioral approach.

However, no intervention integrated all three facets simultaneously. In HF, symptoms are often vague or overlap with other conditions, focusing on awareness alone may not be sufficient [1, 11]. If patients lack the sensitivity to detect subtle changes (interoceptive accuracy), or the natural tendency to attend to bodily sensations (interoceptive sensibility), they may still misinterpret or overlook symptoms, leading to delayed responses and failure of timely medical care-seeking [54, 55]. Recent research supports this view, highlighting that strengthening interoceptive sensibility, may support more accurate symptom perception and timely responses [56]. This suggests that future interventions could benefit from a more integrated approach, focusing on multiple interoceptive facets simultaneously to strengthen patients’ ability to recognize and act upon early signs of HF deterioration.

Several interventions in this review aimed to enhance symptom attribution in patients with HF or CVD. These interventions addressed common barriers of accurate symptom attribution, such as symptom misinterpretation, normalization, and delayed care-seeking. This is consistent with earlier findings that misattribution of symptoms is common among patients with HF [12, 57, 58]. While not all educational interventions explicitly focused on symptom attribution, many included components to enhance symptom recognition, for example through scenario-based training, structured decision aids, or teach back methods. In some studies, these educational components were combined with practical tools such as symptom diaries or daily weight logs, which may help patients integrate symptom monitoring into their daily routines. This combination is in line with broader self-care literature, which suggests that integrating cognitive and behavioral strategies may reinforce patient engagement and skill application in daily life [59, 60]. 

Although interoception and symptom attribution were treated as distinct concepts in this review, a conceptual and practical overlap was observed. Interventions often addressed symptom monitoring and emphasized the importance of timely and appropriate responses to bodily sensations. Interventions focusing on interoception primarily aimed to enhance awareness of internal bodily sensations, whereas those addressing symptom attribution emphasized the cognitive interpretation of symptoms and subsequent behavioral responses. This interrelatedness aligns with the middle-range theory of self-care of chronic illness, which conceptualizes symptom perception as a process involving noticing, interpreting, and responding to bodily changes [6]. Without the initial awareness of symptoms, which depends on interoceptive abilities, accurate interpretation and timely action are unlikely to occur [6, 7]. Interoceptive awareness may thus serve as a foundational element upon which accurate symptom attribution can be built. Interventions that do not address both components may overlook essential aspects of patients’ self-care capacities.

This review primarily included studies targeting patients with HF, and a small number of studies (*n* = 6) involved patients with other CVD. Including CVD studies allowed us for a broader understanding of interventions that could potentially be adapted for HF patients. This broader perspective highlights practical methods and techniques that may inform development of HF-specific interventions, emphasizing opportunities for future research tailored to HF patients.

Finally, although this review did not include whether interventions addressed individual factors such as health literacy, cultural beliefs, or cognitive style, these are recognized in the broader literature as important influences on symptom perception and self-care behaviors [61–63]. Future interventions may benefit from incorporating such personal determinants to enhance relevance and accessibility. Building on this, integrating interoceptive training with symptom attribution strategies may offer a more comprehensive approach to supporting effective self-care in patients with HF.

### Strengths and Limitations

A strength of this review is its broad and inclusive search strategy, which allowed us to identify a wide range of interventions aimed at improving interoception and symptom attribution in patients with HF. By addressing different intervention approaches, this review provides a wide overview of how symptom perception is addressed in different types of intervention. However, there are also several limitations. First, the wide variety in terminology used in literature to describe symptom perception, interoception and attribution, may have resulted in that we missed relevant studies. In some studies, interventions may have addressed symptom perception without explicitly labelling it as such, making them less likely to be included in this review. Second, in some studies, symptom perception was only a small part of a broader intervention, making it difficult to assess the specific contribution on interoception or symptom attribution. Third, the concept of interoception is still developing and used in different ways. Some studies use the term interoception, while others refer to related ideas like body awareness or mindfulness. This overlap and interchangeably use of terms makes it harder to compare studies and draw clear conclusions.

## Conclusion

Interventions on interoception and symptom attribution may help bridge the gap between symptom perception and appropriate self-care for patients with HF. Both interoception and symptom attribution are increasingly addressed through diverse interventions. Interoception was typically addressed through mindfulness-based strategies aimed at increasing awareness of bodily sensations, while symptom attribution was addressed through educational and behavioral approaches supporting interpretation and response to symptoms. In practice, interoception and symptom attribution are closely connected as both contribute to timely and appropriate symptom management. The observed heterogeneity of interventions reflects the complexity of symptom perception in HF and highlights the need for personalized, multicomponent self-care interventions in HF management. Future research could explore how interoception and symptom attribution can be integrated in self-care interventions to enhance symptom perception. Supporting patients in more effectively symptom perception may ultimately contribute to more timely self-care, potentially reducing avoidable hospitalizations and improving quality of life in patients living with HF.

## Key References

- Lee CS, Chu SH, Dunne J, Spintzyk E, Locatelli G, Babicheva V, et al. Body listening in the link between symptoms and self-care management in cardiovascular disease: A cross-sectional correlational descriptive study. Int J Nurs Stud. 2024;156:104809.
  - This paper demonstrates that greater awareness of body sensations and body listening were associated with better self-care management in patients with cardiovascular disease.
- Kleman C, Magnus JM, Andrews M, Meyer K, Lutz BJ. The work of managing a chronic illness: A job description. J Eval Clin Pract. 2023;29(1):166-80.
  - This paper conceptualizes the management of a chronic illness as a form of‘work’, highlighting the cognitive, emotional, and practical demands from patients. Knowing this helps to better identify ways to support patients to maximize patient success in self-care.
- Riegel B, Page SD, Aryal S, Lee CS, Belfiglio A, Freedland KE, et al. Symptom characteristics, perceived causal attributions, and contextual factors influencing self-care behaviors: An ecological daily assessment study of adults with chronic illness. Patient Educ Couns. 2024;123:108227.
  - This study shows that patients’ causal attributions and daily contexts strongly influence self-care behaviors.
- Riegel B, Jaarsma T, Lee CS, Stromberg A. Integrating Symptoms Into the Middle-Range Theory of Self-Care of Chronic Illness. ANS Adv Nurs Sci. 2019;42(3):206-15. doi: 10.1097/ANS.0000000000000237.
  - Expands the middle-range theory of self-care by explicitly integrating symptom recognition and interpretation as central components of chronic illness management.
- Garfinkel SN, Seth AK, Barrett AB, Suzuki K, Critchley HD. Knowing your own heart: distinguishing interoceptive accuracy from interoceptive awareness. Biol Psychol. 2015;104:65–74. doi: 10.1016/j.biopsycho.2014.11.004.
  - Distinguishes interoceptive accuracy from awareness, clarifying how perception and insight into bodily signals differ.

## Data Availability

No datasets were generated or analysed during the current study.
